# Granzyme A in Chikungunya and Other Arboviral Infections

**DOI:** 10.3389/fimmu.2019.03083

**Published:** 2020-01-14

**Authors:** Alessandra S. Schanoski, Thuy T. Le, Dion Kaiserman, Caitlin Rowe, Natalie A. Prow, Diego D. Barboza, Cliomar A. Santos, Paolo M. A. Zanotto, Kelly G. Magalhães, Luigi Aurelio, David Muller, Paul Young, Peishen Zhao, Phillip I. Bird, Andreas Suhrbier

**Affiliations:** ^1^Bacteriology Laboratory, Butantan Institute, São Paulo, Brazil; ^2^QIMR Berghofer Medical Research Institute, Brisbane, QLD, Australia; ^3^Department of Biochemistry and Molecular Biology, Biomedicine Discovery Institute, Monash University, Melbourne, VIC, Australia; ^4^Australian Infectious Disease Research Centre, University of Queensland, Brisbane, QLD, Australia; ^5^Health Foundation Parreiras Horta, Central Laboratory of Public Health, State Secretary for Health, Aracajú, Brazil; ^6^Laboratory of Molecular Evolution and Bioinformatics, Department of Microbiology, Biomedical Sciences Institute, University of São Paulo, São Paulo, Brazil; ^7^Laboratory of Immunology and Inflammation, University of Brasilia, Brasilia, Brazil; ^8^Drug Discovery Biology and Department of Pharmacology, Monash Institute of Pharmaceutical Sciences, Monash University, Parkville, VIC, Australia; ^9^School of Chemistry and Molecular Biosciences, University of Queensland, Brisbane, QLD, Australia

**Keywords:** chikungunya, granzyme A, NK cell, arthritis, arbovirus

## Abstract

Granzyme A (GzmA) is secreted by cytotoxic lymphocytes and has traditionally been viewed as a mediator of cell death. However, a growing body of data suggests the physiological role of GzmA is promotion of inflammation. Here, we show that GzmA is significantly elevated in the sera of chikungunya virus (CHIKV) patients and that GzmA levels correlated with viral loads and disease scores in these patients. Serum GzmA levels were also elevated in CHIKV mouse models, with NK cells the likely source. Infection of mice deficient in type I interferon responses with CHIKV, Zika virus, or dengue virus resulted in high levels of circulating GzmA. We also show that subcutaneous injection of enzymically active recombinant mouse GzmA was able to mediate inflammation, both locally at the injection site as well as at a distant site. Protease activated receptors (PARs) may represent targets for GzmA, and we show that treatment with PAR antagonist ameliorated GzmA- and CHIKV-mediated inflammation.

## Introduction

Granzyme A (GzmA) is a granule trypsin-like serine protease (trypase) secreted by various cytotoxic lymphocytes including NK cells (1, 2), NKT cells (3), CD8 cytotoxic T lymphocytes (CTL) (4), and CD4 CTL (5–7). The established view is that GzmA enters target cells through perforin pores at the immunological synapse (contact site between the cytotoxic lymphocyte and the target cell) to mediate caspase-independent cell death via cleavage of members of the SET complex (1, 8). Although GzmA often remains classified as a cytotoxic mediator (7, 9, 10), an emerging paradigm is that the primary physiological role of GzmA is promotion of inflammation in a variety of settings (11–15). GzmA has, for instance, been implicated as an important proinflammatory mediator in *inter alia* rheumatoid arthritis (16, 17), psoriasis (18), and osteoarthritis (19). A number of mechanisms have been proposed whereby GzmA might mediate this activity, including intracellular cleavage of pro-IL-1β (20) or SET complex proteins (21, 22), and/or extracellular cleavage of pro-urokinase (23) or protease activated receptors 1 and 2 (PAR-1 and PAR-2) (24–27) or potentiation of TLR2/4 (28) and/or TLR9 (29) signaling, with the latter two potentially not requiring GzmA's protease activity. GzmA is also reported to be a critical effector molecule for human Treg function (30). Serpinb6b is a specific inhibitor of mouse GzmA that forms a covalent stoichiometric 1:1 inhibitory complex with GzmA (31). Serpinb6b is upregulated in resolution phase (anti-inflammatory) macrophages in mice (32), perhaps providing further support for the pro-inflammatory role of GzmA. No human equivalent of this serpin has as yet been identified.

Elevated levels of circulating GzmA protein have been observed in a diverse variety of infectious disease settings including viral, bacterial, and parasitic infections (12, 33–37). We recently also showed elevated levels of circulating GzmA in non-human primates infected with chikungunya virus (CHIKV) (38). Circulating mouse GzmA (mGzmA) does not appear to have, or to induce, significant anti-viral activity against CHIKV (38), although anti-viral activity for mGzmA (ostensibly independent of cytolytic activity) has been reported for ectromelia (39). CD8 T cells appear to play only a minor role in CHIKV anti-viral activity and disease (40, 41). In contrast, Th1 CD4 T cells (42) play a major pathogenic role (43–45), with CD56+ (46, 47) NK cells (42, 48, 49) and perhaps NKT cells (50) also contributing (51).

Herein we report that circulating GzmA is significantly elevated in humans and mice following infection with CHIKV, and show that it is also evaluated in mouse models of Zika virus (ZIKV) and dengue virus (DENV) infections. During CHIKV infection in mice, NK cells appear to be the primary source of mGzmA. Injection of recombinant mGzmA was also able to induce edema and neutrophil infiltration in mice. Although the molecular mechanisms that underpin GzmA's pro-inflammatory activities *in vivo* are currently unclear, PAR-1 and PAR-2 may be involved as treatment with PAR-1 and PAR-2 antagonists ameliorated foot swelling induced by recombinant mGzmA. The PAR-1 antagonist, Vorapaxor, was also able to reduce foot swelling after CHIKV infection.

## Materials and Methods

### Human Sera Collection, Diagnosis, and Patient Information

Human serum samples were collected in the Brazilian states of Sergipe, São Paulo, and Brasília (52, 53). Clinical and socio-demographic data was collected through a questionnaire that participants were asked to complete. Patient samples were collected from consented participants reporting arbovirus-like symptoms in the period between 1 and 3 days post the onset of symptoms. qRT PCR tests were undertaken to test for CHIKV, ZIKV, and DENV RNA as described (52). All CHIKV positive patients tested negative for DENV and ZIKV and all the control patients tested negative for CHIKV, ZIKV and DENV.

### Determination of GzmA Levels in Human and Mouse Serum Samples

Human serum samples were tested for human GzmA (hGzmA) levels using the Human Granzyme A Flex Set (BD Cytometric Bead Array, BD Biosciences, San Diego, CA, USA) and Fluorescence-Activated Cell Sorting (FACS) using the Canto II Cell Analyzer (BD Biosciences, San Diego, CA, USA) according to manufacturer's protocols. The data were analyzed with the FCAP Array v 3.0.1 software (BD Biosciences, San Diego, CA, USA).

mGzmA levels were determined using an ELISA kit (MyBioSource, San Diego, CA, USA, MBS704766) according to manufacturer's instructions.

### Mouse Models of CHIKV, ZIKV, and DENV

For the adult wild-type mouse model of CHIKV C57BL/6 female mice 6-8 weeks old were injected with 10^4^ CCID_50_ CHIKV (isolate LR2006 OPY1) s.c. into the feet as described (38, 54). The mouse model of CHIKV-induced hemorrhagic shock using IRF3/7^−/−^ mice has been described previously (55) and involved inoculation with CHIKV as above. The ZIKV_Natal_ strain was used to infect 8-12 week old female IFNAR1^−/−^ mice s.c. (base of tail) with 10^4^ CCID_50_ as described (56, 57). The ZIKV_MR766_ strain was similarly used to infect female IRF3/7^−/−^ mice with 10^3^ CCID_50_. The DENV mouse model used 6-8 week old female AG129 mice infected with 10^5^ pfu DENV-2 (strain D220) i.p. (58). All mice were euthanized using CO_2_ when they reached ethically defined disease severity scores. All work with infectious CHIKV was conducted in the biosafety level-3 (PC3) facility at the QIMR Berghofer Medical Research Institute. All work was approved by the QIMRB Institutional Biosafety Committee.

### Cell Harvesting and FACS Analyses

Feet were removed at the indicated times, kept at 4°C and tissue was scraped from the bone using a scalpel into RPMI 1640 supplemented with 10% fetal calf serum (R10) at 4°C (6-8 feet in 5 mls). The tissue suspensions were digested with collagenase/dispase (Roche, Basel, Switzerland, Cat #10269638001) (1 mg/ml) and DNase I (Roche, Cat#10104159001) (0.2 mg/ml) for 30-45 min at 37°C with occasional mixing. Debris was removed by centrifugation at 10 g for 1 min at 4°C, and the supernatant collected and placed into a new 10 ml tube and underlayed with ≈5 ml Percoll (GE Healthcare, Sweden) and centrifuged at 600 g for 30 min at 4°C. Cells at the Percoll/medium interface were collected and washed once with R10. Red blood cells were lysed with ACK buffer, and the cells were washed twice with R10 at 4°C. Cells were blocked with Fc block (2.4G2 tissue culture supernatant, ≈10 μg/ml) for 20 min 4°C, washed once in phenol red free RPMI supplemented with 2% fetal calf serum (R2) and were then stained with Live/Dead Aqua (Invitrogen, Carlsbad, California, USA, Cat#34957) for 15 min and washed once in R2. Cells were surface stained with anti-NK1.1-BV421 (Biolegend, San Diego, California, USA, clone PK136); anti-CD3-APC (Biolegend, clone 145-2C11); anti-CD4-FITC (Biolegend, clone RM4-5) for 45 min at 4°C. Cells were washed twice in R2 were then fixed and permeabilized with BD Cytofix/Cytoperm for 20 min and washed twice with 1x BD Perm/Wash buffer. Cells were then stained with anti-mouse-GzA-PE (Biolegend, clone 3.8G5) in Perm/Wash buffer for 30 min and then washed twice in Perm/Wash buffer. Cells were than analyzed by FACs using BD LSR Fortessa 4 on the same day. Data was analyzed using BD FACSDiva software v8.0.1. Splenocytes were stained as above with the procedure starting with red blood cell lysis.

### BLT Assay

The benzyloxycarbonyl-L-lysine thiobenzyl ester (BLT) assay was undertaken as described (4) but modified by addition of 2 mM EDTA (to inhibit cysteine proteases) and 1% Igepal (Sigma-Aldrich, St Louis, MO, USA) (instead of NP40 to lyse the cells). The assay was run in duplicate using 2 ×10^5^ NK1.1+ and CD3- cells per well, with cells FACS-sorted from splenocytes of naive C57BL/6J mice. Positive controls were lysates of two human cell lines, CEM T lymphoblast cells (ATCC CRL-2265) and NK92 NK cell line (ATCC CRL-2407), both known to express hGzmA (1).

### Recombinant Mouse GzmA and Serpinb6b

Recombinant mouse GzmA zymogen was produced in *Pichia pastoris* and activated as described previously (59, 60). Production of recombinant Serpinb6b, and GzmA Serpinb6b binding assays were undertaken as described previously (31). mGzmA was injected s.c. into feet of C57BL/6J mice at the indicated dose and foot swelling measured in the injected foot and the contralateral uninjected foot using digital calipers as described (54). Foot swelling was determined as the percentage increased in height x width of the metatarsal area relative to the same foot before injection.

### Drug Treatments

Vorapaxar (SCH 530348) was obtained from Axon Medchem (Groningen, Netherlands) and I-343 was synthesized and purified by Dr. Luigi Aurelio (61). Both drugs were dissolved in dimethylformamide (DMF) (10 mg/ml) and then PBS (0.5% v/v) prior to administration at the indicated dose.

### Statistics

Statistical analysis of experimental data was performed using IBM SPSS Statistics for Windows, Version 19.0. The Spearman correlation was used for hGzmA and CHIKV RNA and disease scores. Two-sample comparison using *t*-test was performed when the difference in variances was <4, skewness was >-2, and kurtosis was <2. Non-parametric data with difference in variances of <4 was analyzed using Mann-Whitney *U*-test, if difference of variances was >4 the Kolmogorov-Smirnov test was employed. The log rank (Mantel-Cox) test was used for statistical analysis of surviving proportions. The repeat measures ANOVA was used for mGzmA-induced foot swelling.

## Results

### Circulating GzmA Levels in CHIKV Patients

Levels of human GzmA (hGzmA) were measured in a cohort of Brazilian patients, who were confirmed to be CHIKV positive by qRT PCR as described (52). The levels were compared to a cohort of healthy CHIKV negative patients. All serum samples from CHIKV patients were obtained between days 1 and 3 post onset of symptoms as reported by the patient. The mean age was 38 ± 17.1 years for CHIKV patients and 43.5 ± 15 years for controls. Of the CHIKV patients, 35 provided their gender as female and 19 provided their gender as male. Of the control patients, 18 provided their gender as female and 5 provided their gender as male. hGzmA levels did not correlate significantly with age (Spearman correlation, *p* = 0.33), nor were differences between males and females significant (*p* = 0.75, *t*-test).

**Figure 1:**
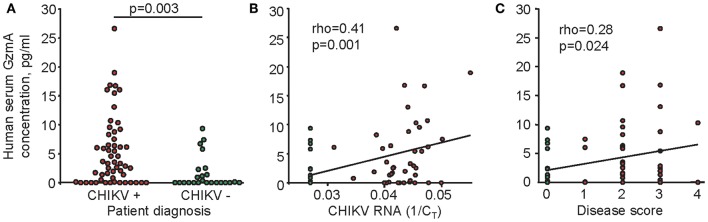
Serum levels of hGzmA in CHIKV and control patients. **(A)** hGzmA concentrations were determined in sera from 56 CHIKV patients (positive by CHIKV qRT PCR and/or serology, red dots) and 24 healthy controls (negative by CHIKV qRT PCR and/or serology, green dots). Statistics by Kolmogorov-Smirnov test. **(B)** Spearman correlation between GzmA concentration and CHIKV RNA levels as determined by qRT PCR. The reciprocal of the C_T_ value (1/C_T_) was taken as a measure of CHIKV RNA levels, with a C_T_ value of 37 deemed to be negative (1/37 = 0.027, green dots, *n* = 24). **(C)** Spearman correlation between hGzmA concentration and disease score. Each of the main symptoms (fever, joint pain, rash, and myalgia) were determined to be present (yes = 1 or no = 0), and the sum of scores (minimum 0, maximum 4) provided the disease score. All healthy controls scored 0 (green dots, *n* = 24).

hGzmA levels were significantly higher in the CHIKV positive group compared to the control group (Figure 1A), confirming the data obtained from CHIKV infected non-human primates (NHP) and a small cohort of Australian travelers (38). The NHP studies illustrated that serum GzmA levels were quite variable even before infection, and increases were transient, peaking between day 2 and 9 post infection (38). Baseline levels were not available for the human cohort and only a single sample post-infection was available for each patient. Differences from baseline could thus not be determined, nor could we match sampling times with peak hGzmA levels. Nevertheless, serum CHIKV RNA levels correlated significantly and positively with hGzmA levels (Figure 1B). (CT values from CHIKV qRT PCR were available for a number of patients, with relative CHIKV RNA levels nominally taken as the reciprocal of the CT values). A similar correlation was apparent from NHP studies (38). Importantly, hGzmA levels also correlated positively with disease scores (Figure 1C), a key parameter that was not available from NHP studies. Although control subjects in the cohort were overtly healthy at the time that blood was taken, one control patient (hGzmA level 6.76 pg/ml) reported a past infection with DENV and was taking a thyroid hormone supplement (62). Clinical histories were not provided by many patients and controls.

### Serum GzmA Levels in CHIKV-Infected Wild-Type Mice

**Figure 2:**
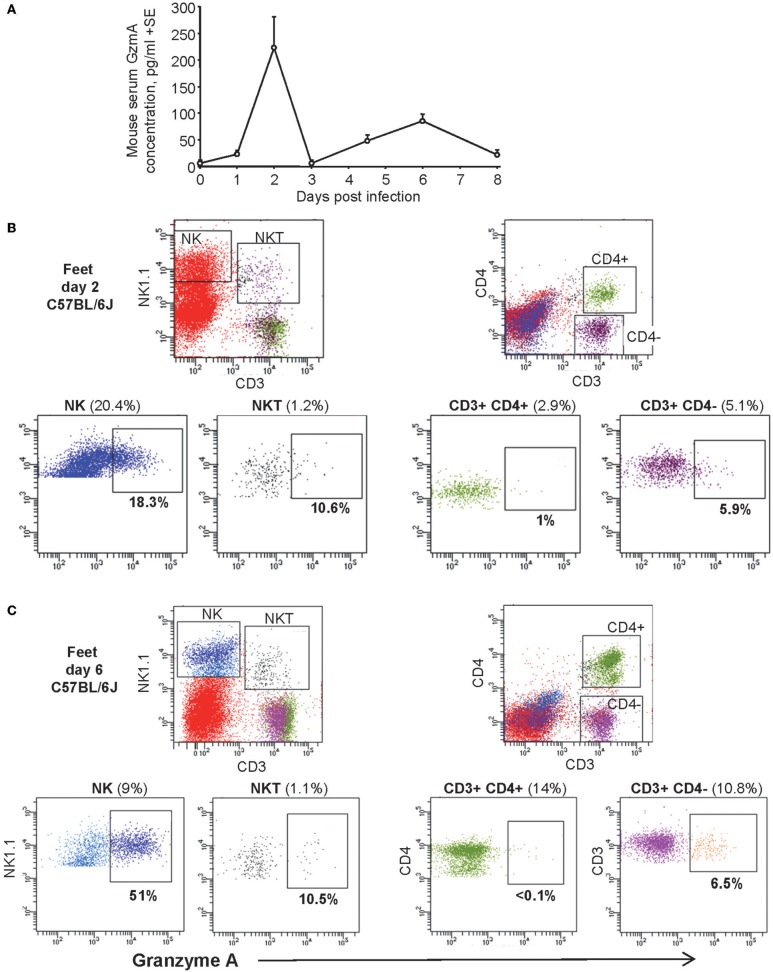
Serum GzmA levels in CHIKV-infected adult wild-type mice. **(A)** C57BL/6 mice were infected with CHIKV and serum analyzed for GzmA by ELISA. Data from two independent experiments with 6-12 mice per time point. **(B)** Cells isolated from feet (*n* = 6-8) from CHIKV-infected C57BL/6 mice were pooled and analyzed by FACS for intracellular GzmA expression on day 2 post infection. Percentages in brackets are the % of total live isolated cells from the feet. Percentages below the boxes are the percent of the indicted cell type that are GzmA positive. **(C)** Cells analyzed and gated as for B using cells isolated from feet 6 days post infection with CHIKV.

The levels of mouse GzmA (mGzmA) in serum were determined in an adult wild-type mouse model of CHIKV infection (54). This mouse model has been shown to recapitulate many aspects of human infection, inflammatory responses and disease (38, 54, 63). Two peaks in serum mGzmA levels were apparent on day 2 (≈200 pg/ml) and day 6 (≈75 pg/ml) (Figure 2A), which coincide with peak viremia and peak arthritis (38, 54). These two peaks also coincide with the two peaks in foot swelling seen in this model (54) (i) the smaller peak in foot swelling on day 2-3, which is edematous and may be associated with NK cell activity (42, 48) and (ii) the larger peak on day 6-7, which is associated with a pronounced mononuclear cellular infiltrate comprising primarily monocytes/macrophages, NK cells and T cells (54).

### FACS Analyses of mGzmA Expressing Cells After CHIKV Infection

To ascertain which cells produce mGzmA during CHIKV infection in the adult wild-type C57BL/6J mouse model, FACS analyses were undertaken using intracellular staining for mGzmA (2). In resting splenocytes about 80% of NK1.1+, CD3− cells (NK cells) expressed mGzmA protein in our assays (Supplementary Figure 1A), consistent with previous findings (2). The specificity of the intracellular mGzmA staining was demonstrated by <0.1% of cells from GzmA^−/−^ mice staining with anti-mGzmA antibody (Supplementary Figure 1C).

On day 2 post CHIKV infection the cells infiltrating into the feet (54) comprised ≈20% NK cells (Figure 2, NK1.1+, CD3−), with ≈18% of these expressing detectable mGzmA (Figure 2B). The proportion of NKT cells (NK1.1+, CD3+), CD4+ CD3+ cells (CD4 T cells) and CD4− CD3+ cells (primarily CD8 T cells) were low (<5.1%), with mGzmA expression seen in 10.6% of NKT cells and 5.9% of CD4− CD3+ cells (Figure 2B). On day 6 post CHIKV infection the proportion of NK cells had dropped to 9%, with 51% of these cells expressing detectable mGzmA (Figure 2C). The proportion of CD4+ CD3+ cells increased by ≈5 fold and the proportion of CD4− CD3+ cells doubled, but the percentage of these cell expressing mGzmA did not change (Figure 2C). FACS controls using GzmA^−/−^ mice are shown in Supplementary Figure 2.

Thus, on both day 2 and 6 post CHIKV infection, the major source of circulating mGzmA would appear to be NK cells, with NK cells involved in arthritic immunopathology (and perhaps anti-viral activity) (42, 48, 50, 64). As 80% of NK cells in the spleen are mGzmA+, one might speculate that the reduction in the percentage of mGzmA+ NK cells in feet on day 2 (20.4% with 18.3% mGzmA+) and on day 6 (9% with 51% mGzmA+) indicates NK cell degranulation, as these figures would be consistent with the high levels of circulating mGzmA on day 2 and the lower peak on day 6 (Figure 2A). However, these FACS results (Figures 2B,C) could conceivably also arise from preferential recruitment of mGzmA-negative NK cells into feet. FACS analyses of splenocytes suggest migration of NK cells out of the spleen by day 2 post infection (as the proportion of NK cells drops from 2 to 1.1%) and subsequent expansion of NK cells (65) by day 6 (with the proportion increasing to 5.4%) (Supplementary Figure 1B).

Although the CD4 T cells recruited into the arthritic lesions are predominantly Th1 biased and CD4 T cells are major drivers of arthropathy (40, 43, 45, 51), they do not appear to be a major source of mGzmA. CD4− CD3+ cells, primarily CD8 T cells, would be expected to express mGzmA (4); however, CD8 T cells appear to neither have significant protective activities nor immunopathological roles in alphaviral arthritides (40, 66). NKT cells might be expected to express GzmA (67) and they may play a role in CHIKV infections, although their relative importance remains to be established (68).

### The BLT Assay and Recombinant Murine GzmA

**Figure 3:**
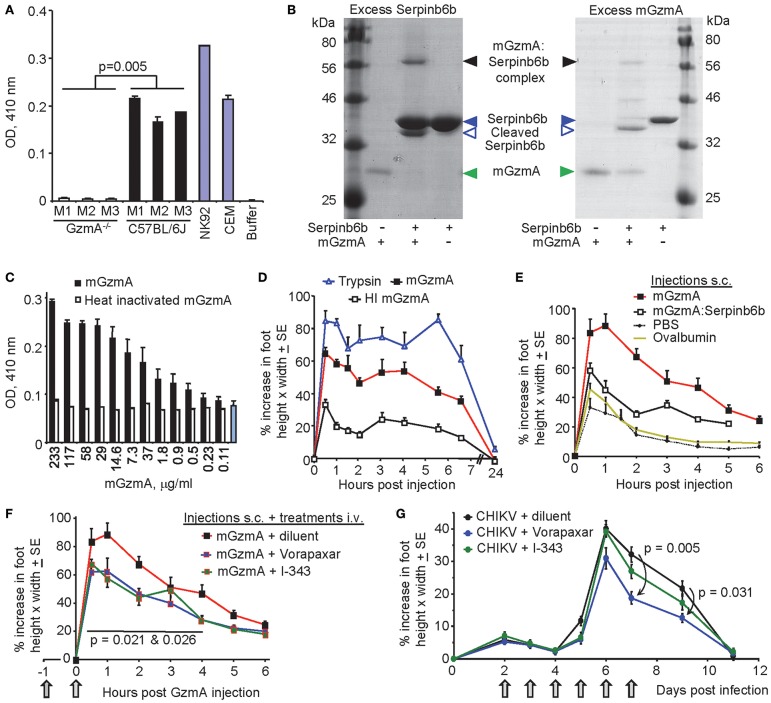
Activity and bioactivity of mouse GzmA. **(A)** FACS-sorted NK cells from resting spleens were lysed, and lysates measured for BLT esterase activity in duplicate for 3 GzmA^−/−^ mice and 3 C57BL/6J mice. NK92 and CEM cell lines constitutively express GzmA and lysates from these lines were used as positive controls. Statistics by Kolmogorov Smirnov test using all replicates. **(B)** Recombinant mouse GzmA (mGzmA) and recombinant Serpinb6b were incubated together (37°C for 20 min) either with a 6-fold molar excess of Serpinb6b (Excess Serpinb6b) or a 3-fold molar excess of mGzmA (Excess mGzmA). mGzmA, Serpinb6b, and complexes were then resolved by SDS-PAGE and stained with Coomassie Brilliant Blue. Some breakdown products of the complex are evident in Excess mGzmA. **(C)** BLT activity of mGzmA and heat-inactivated mGzmA. The indicated concentrations of recombinant mGzmA and heat inactivated mGzmA were tested in duplicate in a BLT assay. The blue bar represents background BLT activity in the absence of any protein. **(D)** Injection (10 μl) s.c. into feet of recombinant mGzmA (5 μg), heat inactivated (100°C, 1 h) mGzmA (5 μg), or trypsin (20 μg) (*n* = 6 mice per group). **(E)** Injection (30 μl) s.c. into feet of mGzmA (5 μg), mGzmA (5 μg) complexed with 3.9 μg recombinant Serpinb6b (2:1 molar ratio, 1 h 37°C), ovalbumin (8.9 μg) or PBS (*n* = 6 mice per group). **(F)** The same experiment shown in E with two extra groups where mice injected with mGzmA were also treated at the indicated times (gray arrows) with Vorapaxar (20 μg/mouse i.v. twice) or I-343 (20 μg/mouse twice i.v.). The diluent control treatment was with DMF/PBS i.v. Repeat measure ANOVA from 0.5 to 4 h relative to diluent control Vorapaxar *p* = 0.026, I-343 *p* = 0.021. **(G)** Mice were infected with CHIKV as in Figure 2A and were treated with Vorapaxar or I-343 (10 μg/mouse i.v. daily for 6 days) or diluent. The right and left foot was averaged for each mouse. Statistics by Kolmogorov Smirnov tests (*n* = 6 mice per group).

GzmA is ordinarily stored in granules as a mature protease, with the low pH of the granule preventing (premature) proteolytic activity (69). hGzmA's activity can be determined via the protease's esterase activity using the benzyloxycarbonyl-L-lysine thiobenzyl ester (BLT) assay (4). NK cells were FACS sorted from the splenocytes of C57BL/6J mice (NK1.1+, CD3−, >98% pure) and detergent lysates (pH = 8.1) subjected to analysis using the BLT assay. NK cells from C57BL/6J mice, but not NK cells from GzmA^−/−^ mice, showed significant BLT activity (Figure 3A). This confirms that resting splenic NK cells from C57BL/6J mice contain enzymatically active mGzmA (2) and illustrates the utility of the BLT assay for measuring mGzmA activity.

Recombinant mGzmA and recombinant mouse Serpinb6b were generated as described previously (31, 70). Purity and activities are illustrated by Coomassie staining (Figure 3B). When recombinant mGzmA was incubated with excess SerpinB6b, all the mGzmA was found in the complex (Figure 3B, Excess Serpinb6b), illustrating that the majority of mGzmA was correctly folded and able to bind the inhibitor. When recombinant Serpinb6b was incubated with excess mGzmA, >95% was found in the complex or was cleaved in the reactive center loop (Cleaved Serpinb6b) (Figure 3B, Excess mGzmA), illustrating that most of the recombinant Serpinb6b was correctly folded and able to bind mGzmA. The recombinant mGzmA was also shown to be active in the BLT assay with heat inactivation destroying the BLT activity (Figure 3C), indicating that the recombinant mGzmA was enzymically active.

### Proteolytically Active GzmA Alone Is Sufficient for Inflammation Induction

Injection of proteases subcutaneously (s.c.) into the feet of mice has been used as an assay to evaluate their pro-inflammatory activities (71–74). Injection of recombinant mGzmA s.c. into mouse feet resulted in significant foot swelling when compared with injection of heat-inactivated mGzmA; trypsin was used as a positive control (71) (Figure 3D). This experiment was repeated with 2 additional controls (Figure 3E); (i) injection of ovalbumin (an equivalent μg dose of a proteolytically inactive foreign protein), which induced no increase in foot swelling over PBS, illustrating that the foot swelling was not simply due to injection of protein and (ii) injection of mGzmA complexed with Serpinb6b, which induced substantially lower foot swelling than mGzmA, indicating that the (proteolytically inactive) Serpinb6b:mGzmA complex had limited proinflammatory activity in this assay.

Interestingly injection of recombinant mGzmA into one foot resulted in slight, but significant and similarly rapid, swelling in the contralateral foot (which had received no injections) (Supplementary Figure 3A). This suggested some injected mGzmA reached the other foot via the circulation and induced inflammation at the distant site.

### Treatment With Protease Activated Receptor 1 and 2 Antagonists

GzmA has been reported to cleave PAR-1 (also known as the thrombin receptor) (24) and has been implicated in PAR-2 cleavage (26). Both PAR-1 and PAR-2 have been implicated in exacerbation of arthropathy (75, 76) and promotion of inflammation (74, 77). Mice injected subcutaneously with recombinant mGzmA were thus treated with the PAR-1 agonist Vorapaxar (78) and the PAR-2 antagonist I-343 (61). Both drug treatments provided a significant reduction in foot swelling in feet injected with mGzmA (Figure 3F). Vorapaxar and I-343 treatment also inhibited swelling of the contralateral feet (Supplementary Figure 3A). These data suggest that the pro-inflammatory activity of mGzmA involves (either directly or indirectly) PAR-1 and PAR-2. Note both drugs were dissolved in dimethylformamide (DMF) rather than dimethyl sulfoxide (DMSO), to avoid the potentially confounding anti-inflammatory activity of DMSO (79).

Vorapaxar and I-343 were also used to treat CHIKV arthritis. Treatment was started on day 2 post infection to minimize any effects on viraemia, which peaks at this time (54). Significant reductions in foot swelling were apparent on days 7 and 9 for Vorapaxar (Figure 3G), suggesting that PAR-1 is involved in CHIKV-induced inflammatory arthropathy. A repeat experiment is shown in Supplementary Figure 3B.

### Histology and Immunohistochemistry

**Figure 4:**
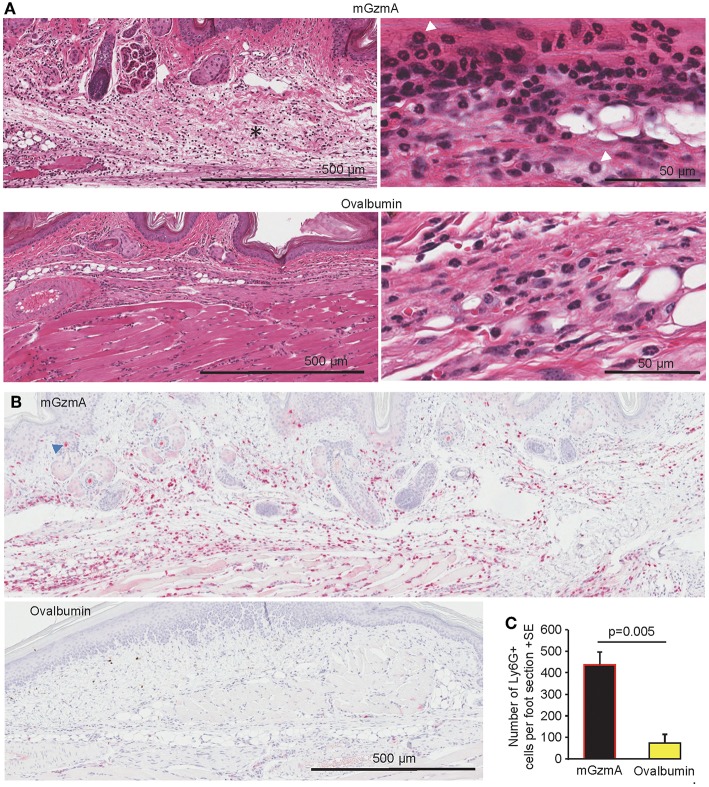
Histology and IHC. **(A)** H&E staining of feet sections taken 4 h after injection of mGzmA or ovalbumin (as in Figure 3E). *indicates subcutaneous oedema surrounded by infiltrating cells. High resolution images are shown on the on the right. White arrowheads indicate examples of cells with polymorphonuclear morphology. **(B)** IHC staining with anti-Ly6G of foot sections taken 4 h after injection of mGzmA or ovalbumin (as for **A**). Neutrophils stain dark red (Warp Red). Pale red staining of hair follicles (blue arrowhead) is artifactual. **(C)** Quantitation of the IHC staining using 5/6 feet from 5/6 mice per group and the mean of 2/3 sections per foot. Statistics by Kolmogorov-Smirnov test (*n* = 5/6 per group).

Histology was undertaken on feet 4 h after s.c. injection of recombinant mGzmA or ovalbumin. H&E staining shows oedema and cellular infiltrates after mGzmA injection, which were substantially less apparent after ovalbumin injection (Figure 4A). Most of the infiltrating cells had a polymorphonuclear morphology (Figure 4A, top right). Immunohistochemical (IHC) staining with anti-Ly6G [a neutrophil specific marker (80)] revealed a higher number of neutrophils in the mGzmA group when compared to the ovalbumin group (Figure 4B). Quantitation of this IHC staining showed high significance (Figure 4C). (F4/80 staining was not significantly different, data not shown).

### GzmA Levels in Interferon-Deficient Mouse Models of Arboviral Infections

**Figure 5:**
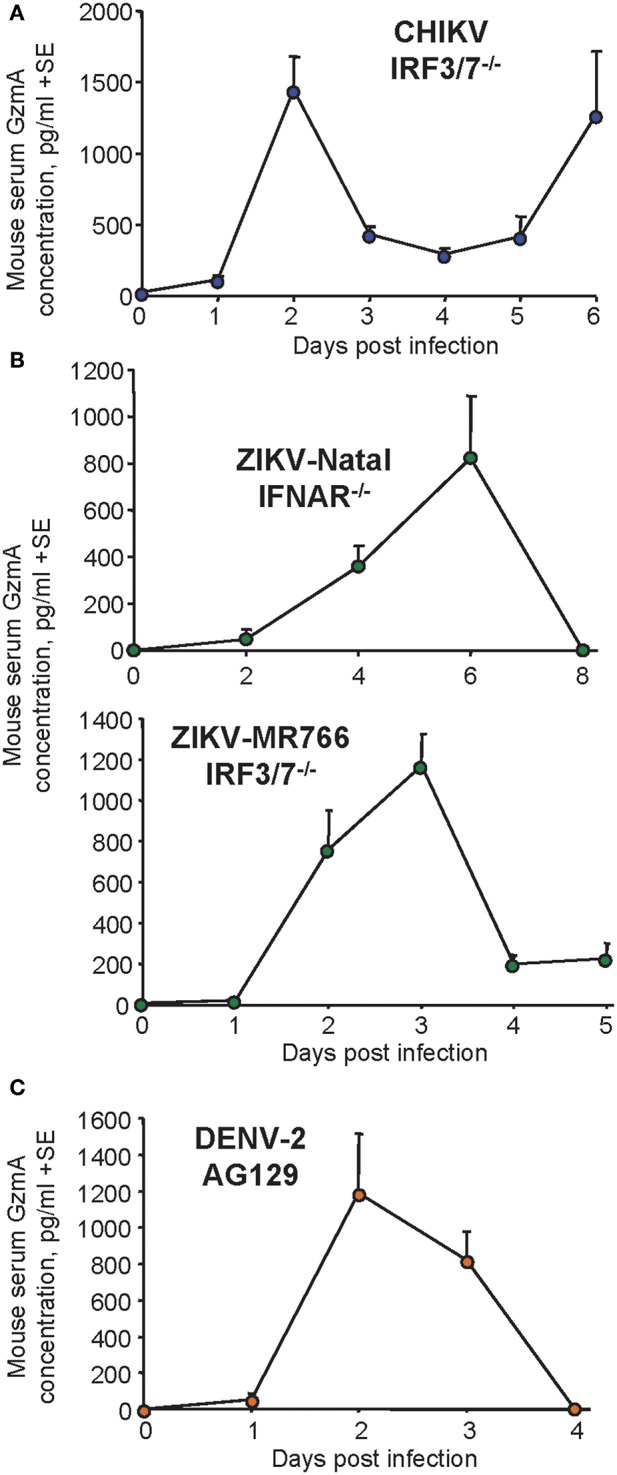
Serum GzmA levels in type I IFN deficient mouse models of CHIKV, ZIKV and DENV **(A)** IRF3/7^−/−^ mice were infected with CHIKV and serum analyzed for mGzmA by ELISA. Infection is lethal with mice euthanized day 5-6. Data from two independent experiments, with 6-12 mice per time point, except day 6 (*n* = 4; with 8 of 12 mice reaching ethically defined criteria requiring euthanasia). **(B)** IFNAR^−/−^ mice (*n* = 4) were infected with ZIKV-Natal (Asian genotype) and IRF3/7^−/−^ mice (*n* = 6) were infected with 10^4^ CCID_50_ ZIKA-MR766 (African genotype) and serum samples analyzed for mGzmA levels. **(C)** AG129 mice (*n* = 5) infected with DENV-2 (D220), and serum samples analyzed for mGzmA levels.

CHIKV infection of mice deficient in interferon response factors 3 and 7 (IRF3/7^−/−^) provide a model of CHIKV hemorrhagic shock, with mice showing high viraemia (peaking on day 3 post infection), cytokinemia (high IFNγ, TNF, IL-6, peaking on day 2), fever (day 2), hypothermia (day 4-5), oliguria (day 4-5), thrombocytopenia (day 3-5), raised hematocrits (day 5), hemorrhage and mortality on day 4-6. GzmA levels peaked on day 2 post infection, reaching high levels (range ≈500–3,000 pg/ml) that were on average ≈7 fold higher in IRF3/7^−/−^mice (Figure 5A) than those seen in wild-type mice (Figure 2A). The increase on days 5/6 may be associated with the hemo-concentration associated with hemorrhagic shock (55). mGzmA was again largely associated with NK cells in this model (Supplementary Figure 4). [Only splenocytes were available for analysis, as foot swelling in this model is not associated with a significant cellular infiltrate (55)]. Treatment with Serpinb6b did not provide significant protection against either foot swelling or mortality in this IRF3/7^−/−^ model of hemorrhagic shock (Supplementary Figure 5).

Two ZIKV isolates ZIKV_Natal_ (an Asian genotype virus from Brazil) and ZIKV_MR766_ (a virulent African genotype virus) (57, 81, 82) were used to infect type I interferon receptor deficient (IFNAR^−/−^) mice and IRF3/7^−/−^ mice (83), respectively. Neither infection is lethal, with viraemia peaking day 2-3 post infection (at a mean of ≈3 log_10_CCID_50_/ml) for ZIKV_Natal_ (57) and day 2 post with a mean peak viraemia of ≈10^5^ CCID_50_/ml for ZIKV_MR766_ (56). High levels of mGzmA were seen, reaching means of ≈800 and ≈1,200 pg/ml, although the peak occurred later on day 6 for the less virulent ZIKV_Natal_ (Figure 5B).

To investigate serum mGzmA levels in DENV infections, the well-established AG129 mouse model was used (58, 84). These mice have no type I or type II interferon receptors and inoculation with DENV-2 (strain D220, 10^5^ PFU, i.p.) results in viremia peaking day 2-3 (at 10^4^–10^5^ pfu/ml), with euthanasia required around day 5. On day 4 post infection animals exhibit significant vascular leakage (with limited hemorrhage) in several tissues (58). Early large increases in circulating mGzmA (reaching a mean of ≈1,200 pg/ml) were again observed (Figure 5C).

## Discussion

We show here that circulating hGzmA levels are elevated during CHIKV disease in humans and that levels correlate with both viral load and disease severity. Using mouse models, we show that NK cells are the major source of mGzmA, consistent with other studies in mice (33, 85). CD56+ NK cells have also been described in CHIKV patients (46, 47) and human CD56^hi^ NK cells have been shown to express high levels of hGzmA, with relatively low levels of perforin (19, 86). NK cells are part of the early innate anti-viral response to many virus infections (65), consistent with the early rise in serum hGzmA and mGzmA described herein. In wild-type mice infected with CHIKV, significant numbers of NK cells were also present in the early infiltrate day 2 post infection. Although NK cells have well-established anti-viral activity (65), mGzmA does not appear to mediate significant anti-viral activity against CHIKV (38). Nevertheless, NK cells have been implicated in inflammatory immunopathology in several settings (87–89), including CHIKV arthropathy (48) where mouse models suggest they may promote oedema (42). A pro-inflammatory role for NK-derived mGzmA has also been implicated in bacterial sepsis (90), with viral sepsis a rare but potentially fatal complication of acute CHIKV infection in humans (91).

The hGzmA levels reported herein ranged from 0 to 27 pg/ml (mean of 5.4 ± SD 6) in a cohort of 56 Brazilian CHIKV patients, and ranged from 0 to 180 pg/ml (mean of 39.1 ± SD 70.9) in a previously reported small (*n* = 6) CHIKV patient cohort comprising Australian visitors returned from overseas (38). GzmA levels in NHPs showed peak levels ranging from 80 to 370 pg/ml (*n* = 9) (38). In C57BL/6 mice, peak levels on day 2 ranged from 40 to 554 pg/ml (averaging at ≈220 pg/ml) (*n* = 12) (Figure 2A). hGzmA was reported to be present in the sera of 98 patients with dengue fever at a median level of 282 pg/ml (range 56–5,058), with healthy controls showing median levels of 15 pg/ml (range 3–124) (35). The latter study used an in-house ELISA and standards. hGzmA levels in CHIKV patients would thus appear to be somewhat lower compared with CHIKV animal models and DENV patients. However, reliable comparisons need to await (i) time series analyses post CHIKV infection to capture peak levels of hGzmA and (ii) formal cross-species validation of the relative performances of the different GzmA ELISA kits. Whether the aforementioned circulating serum concentrations of GzmA mediate significant or substantial bioactivity remains to be established. The ability of subcutaneously injected recombinant mGzmA to mediate swelling on the contralateral foot, nominally argues that <5 μg/ml of mGzmA (5 μg injected, with a mouse blood volume of ≈1 ml) is systemically overtly bioactive, with this concentration reportedly seen in some DENV patients (35).

Circulating hGzmA appears to remain largely proteolytically active (92). PAR-1 and PAR-2 cleavage by mGzmA have been implicated herein and elsewhere as a mechanism whereby mGzmA promotes inflammation (24, 26). However, we are currently undertaking detailed molecular studies to determine if GzmA cleaves PAR-1 and/or PAR-2 under physiological conditions, rather than PAR-1 and/or PAR-2 being involved somewhere in the GzmA- or CHIKV-induced pro-inflammatory cascades. There are >550 proteases in the mouse and human genomes (93), with PAR1/2 cleaved by many different inflammation-associated proteases (77) including granzyme K (38, 94, 95). PAR1/2 have also been shown to be involved in a range of mouse models of arthritis (76, 96–98), suggesting their widespread involvement in this type of immunopathology. The role of neutrophils in mGzmA-mediated foot swelling and its inhibition by PAR1/2-antagonists is consistent with the known involvement of PAR-1 and PAR-2 in neutrophil-associated inflammation (73, 99, 100). However, alphaviral arthritides generally have few neutrophils (51, 101, 102) arguing that other factors are in play, or that the presence of other proteases or inflammatory activities modulate mGzmA activity during CHIKV arthritis. Unraveling the role of PAR1/2 is also complicated by the ability of certain proteases [including hGzmA (25)] to induce biased signaling, whereby some but not other inflammatory pathways are activated (72, 74, 103, 104). In addition, there are distinct differences between mGzmA and hGzmA (31, 105), with mice (but not humans) also encoding the specific mGzmA inhibitor (Serpinb6b) which is expressed by resolution phase macrophages during CHIKV arthritis (101).

What might be the physiological function (if any) of the rapid early post-infection rise in circulating GzmA? As two other granule components, perforin and GzmB, can also be found in the circulation (106, 107), serum GzmA may simply represent a by-product of the anti-viral responses of activated NK cells and other cytotoxic lymphocytes. Circulating GzmA could thus be viewed as a systemic biomarker for cytotoxic lymphocyte activity somewhere in the body. However, we show herein, for the first time, that injection of purified recombinant mGzmA was able to mediate acute inflammation, both locally and in the contralateral foot, arguing that circulating GzmA may function to distribute proinflammatory activity systemically (12, 15). Conceivably, circulating GzmA might act as an “danger signal” or alarmin (29), providing systemic notification (in the current setting) of the engagement of NK cells with arbovirus infected cells. Increased circulating mGzmA levels in IFN deficient mice may thereby reflect increased “danger,” given the lack of protective IFN activities and high viral loads. The lack of significant inflammatory activity mediated by the mGzmA:Serpinb6b complex argues that mGzmA's protease activity is required for its pro-inflammatory activity. This argument would clearly be strengthened if we had a better understanding of the molecular mechanism(s) responsible for GzmA's pro-inflammatory activity.

Th1 CD4 T cells are thought to be the major drivers of CHIKV arthritic disease (40, 43, 51). GzmA-expressing CD4 CTL have been identified in several viral infections (HIV, CMV, vaccinia, DENV) (6, 108) and in rheumatoid arthritis (6). However, we found no evidence that CHIKV infection induces expression of mGzmA in CD4 T cells. To the best of our knowledge, there are no studies showing CHIKV-specific CD4 T cells to be cytotoxic or to express significant levels of GzmA. GzmA secretion by Th1 CD4 T cells is thus unlikely to be a major driver of CHIKV arthritis. Conceivably, NK-derived GzmA might promote CD4 T cell activation via activation of antigen presenting cells (20, 29, 109).

The ability of Vorapaxar to inhibit CHIKV-induced foot swelling might argue that PAR-1 is a potential new target for anti-inflammatory treatment of alphaviral arthritides. However, in humans Vorapaxar is generally used to inhibit PAR-1 on platelets (110), with PAR-1 not expressed on mouse platelets. Given that hemorrhagic manifestations are uncommon, but well-documented, during acute CHIKV infections, platelet inhibition would not be recommended; especially if there was a possibility that the patient had a DENV infection (51). Even if biased PAR-1 antagonists (74, 103) could be developed that did not inhibit platelet function (25), the very rapid early rise in GzmA levels post-infection likely also leaves an unrealistically narrow window between diagnosis and treatment initiation. Given the role of PAR-2 in persistent pain (61) another avenue potentially worthy of investigation is the treatment of persistent CHIKV arthralgia (51) with PAR-2 antagonists; however, an animal model in which chronic CHIKV joint pain can be readily monitored has yet to be developed.

In summary raised levels of circulating GzmA are evident in acute infections of medically important arboviruses, and recombinant mGzmA was by itself able to mediate inflammation. Although PAR-1 and PAR-2 antagonists appear, at least partially, to inhibit foot swelling induced by mGzmA injection, a physiological role for GzmA in direct PAR1/2 cleavage and signaling has yet to be established.

## Data Availability Statement

All datasets generated for this study are included in the article/Supplementary Material.

## Ethics Statement

For samples collected in Sergipe and São Paulo, human studies were approved by the ethics committee (CEPSH/ICB) of the Instituto de Ciências Biomédicas da Universidade de São Paulo (ICB-USP) (Authorization #1284/CEPSH-CAAE: 54937216.5.0000.5467 e CAAE: 61551116.3.0000.5553). The samples are part of a registered biorepository under the custody of PZ, who authorized their use in the studies described herein. For samples collected in Brasília, human studies were approved by the Ethics Committee of Secretaria de Estado de Saúde do Distrito Federal (FEPECS/SES/DF) (CAAE: 36249214.0.0000.5553). The samples were part of the Laboratório Central de Saúde Pública do DF (LACEN-DF) biorepository and permission for their use for measuring granzyme levels was provided by LACEN-DF. All serum sample donors signed a consent form. Mouse work was conducted in accordance with the Australian code for the care and use of animals for scientific purposes as defined by the National Health and Medical Research Council of Australia. Mouse work was approved by the QIMR Berghofer Medical Research Institute animal ethics committee (P2195, A1604-611M, and P1060 A705603M). Dengue work was also approved by the University of Queensland Animal Ethics Committee.

## Author Contributions

ASS, DB, CS, PMAZ, and KM: obtained the clinical samples and data and undertook the human analyses. TL, DK, CR, and NP: undertook the other experiments. LA, DM, and PY: provided vital reagents and samples. PZ, PB, NP, and AS: funding acquisition. ASS, AS, and PB: conceptualized the study. AS: wrote the manuscript with input from PB, ASS, TL, and NP.

### Conflict of Interest

The authors declare that the research was conducted in the absence of any commercial or financial relationships that could be construed as a potential conflict of interest.
