# Supplementary material for: Granzyme A in Chikungunya and Other Arboviral Infections
Source: Front Immunol. 2020 Jan 14;10:3083. doi: 10.3389/fimmu.2019.03083 (PMC6971054; doi:10.3389/fimmu.2019.03083)
Supplement: Supplementary file 1 [file Presentation_1.PPTX]

## Slide 1
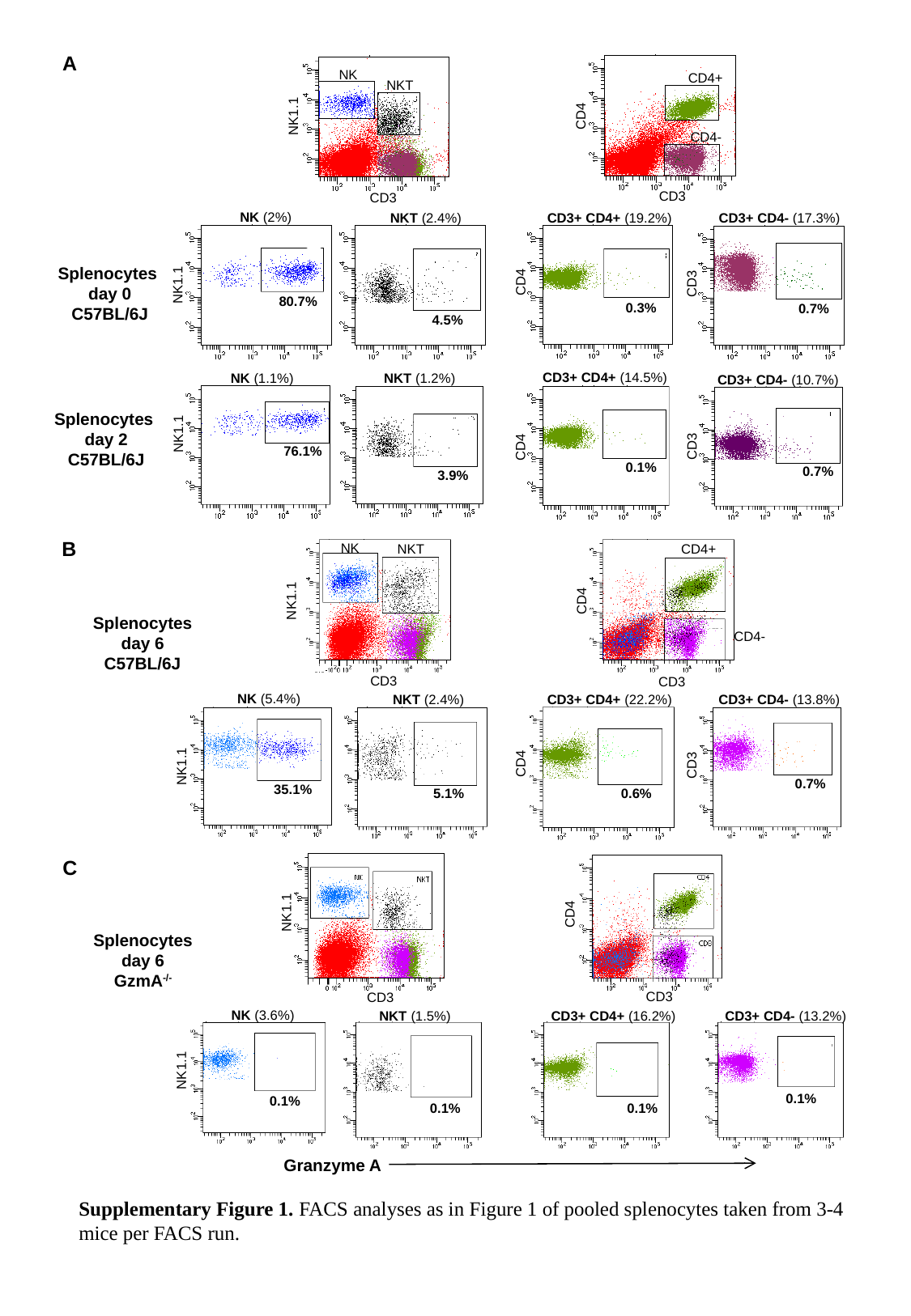

A
NK
CD4+
NKT
NK1.1
CD4
CD4-
CD3
CD3
NK (2%)
CD3+ CD4- (17.3%)
NKT (2.4%)
CD3+ CD4+ (19.2%)
Splenocytes
day 0
C57BL/6J
CD4
CD3
NK1.1
80.7%
0.3%
0.7%
4.5%
CD3+ CD4+ (14.5%)
NK (1.1%)
NKT (1.2%)
CD3+ CD4- (10.7%)
Splenocytes
day 2
C57BL/6J
NK1.1
CD3
CD4
76.1%
0.1%
0.7%
3.9%
B
NK
NKT
CD4+
CD4-
NK1.1
CD4
Splenocytes
 day 6
C57BL/6J
CD3
CD3
NK (5.4%)
CD3+ CD4- (13.8%)
NKT (2.4%)
CD3+ CD4+ (22.2%)
CD4
CD3
NK1.1
0.7%
35.1%
5.1%
0.6%
C
NK1.1
CD4
Splenocytes
 day 6
GzmA-/-
CD3
CD3
NK (3.6%)
CD3+ CD4- (13.2%)
NKT (1.5%)
CD3+ CD4+ (16.2%)
NK1.1
0.1%
0.1%
0.1%
0.1%
Granzyme A
Supplementary Figure 1. FACS analyses as in Figure 1 of pooled splenocytes taken from 3-4 mice per FACS run.

## Slide 2
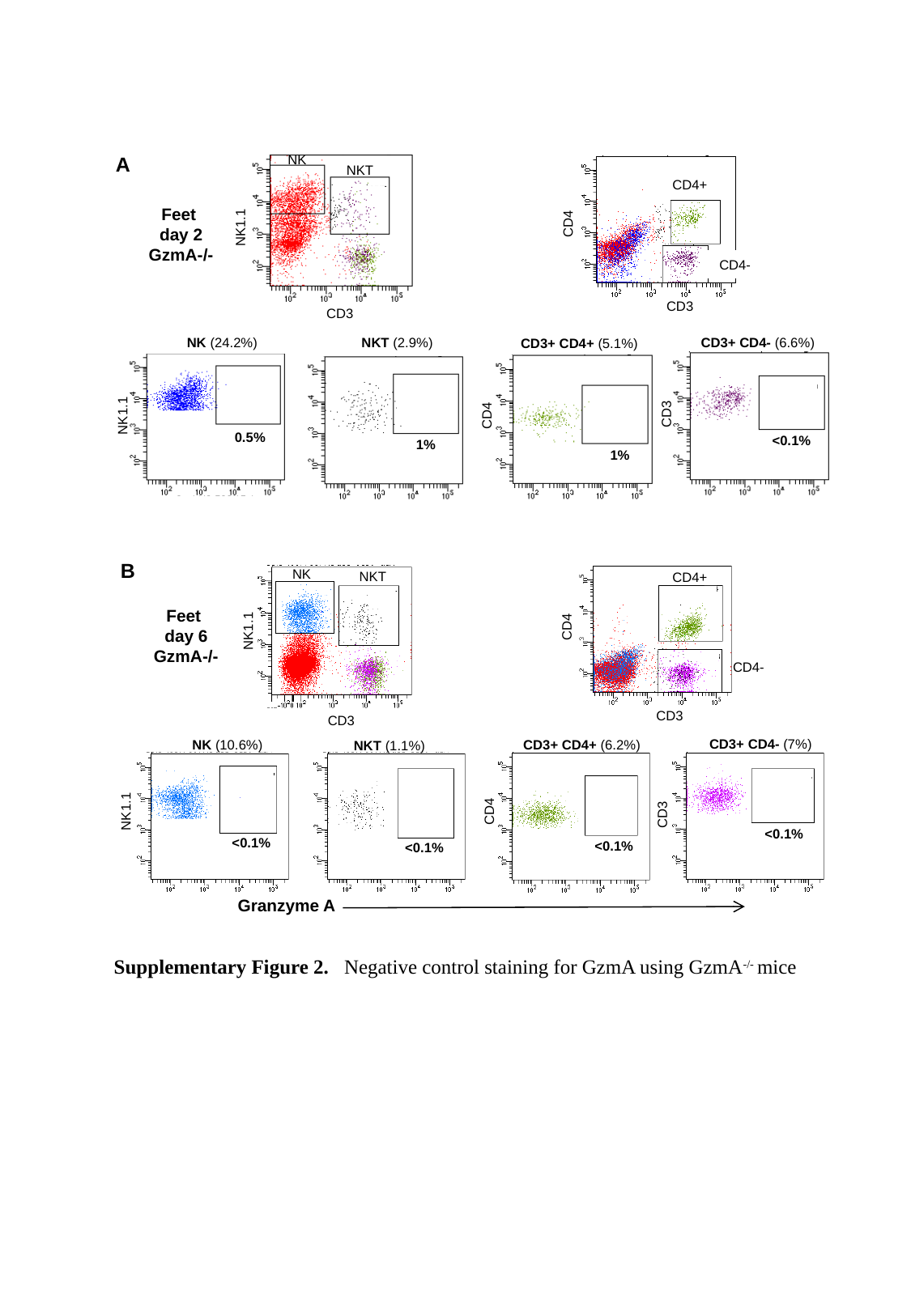

NK
A
NKT
CD4+
Feet
day 2
GzmA-/-
CD4
NK1.1
CD4-
CD3
CD3
NK (24.2%)
CD3+ CD4- (6.6%)
NKT (2.9%)
CD3+ CD4+ (5.1%)
CD3
NK1.1
CD4
0.5%
<0.1%
1%
1%
B
NK
NKT
CD4+
Feet
day 6
GzmA-/-
CD4
NK1.1
CD4-
CD3
CD3
CD3+ CD4- (7%)
CD3+ CD4+ (6.2%)
NK (10.6%)
NKT (1.1%)
NK1.1
CD4
CD3
<0.1%
<0.1%
<0.1%
<0.1%
Granzyme A
Supplementary Figure 2. Negative control staining for GzmA using GzmA-/- mice

## Slide 3
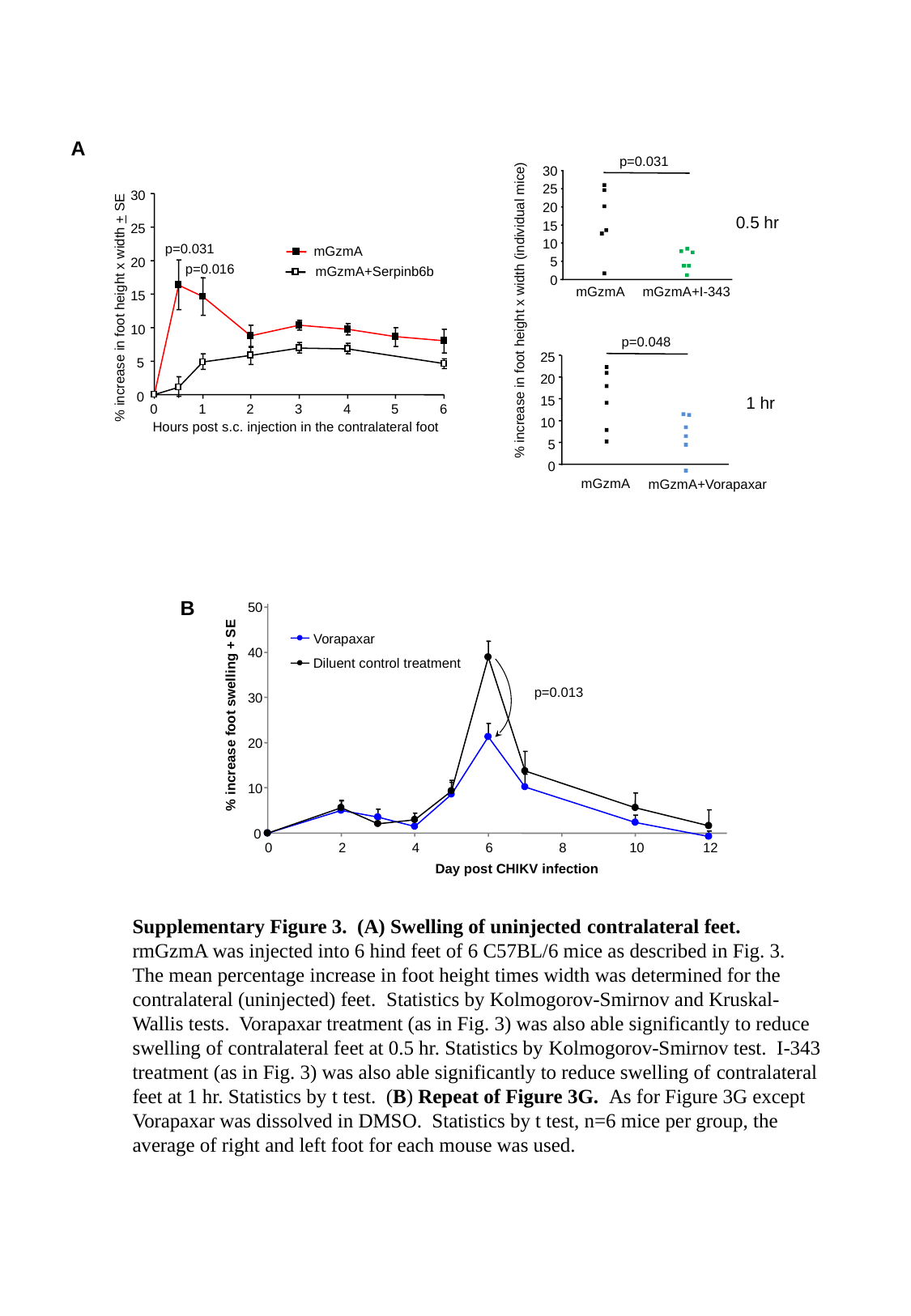

A
p=0.031
30
25
30
25
20
15
10
5
0
20
0.5 hr
15
p=0.031
10
mGzmA
5
p=0.016
mGzmA+Serpinb6b
0
mGzmA
mGzmA+I-343
 % increase in foot height x width + SE
 % increase in foot height x width (individual mice)
p=0.048
25
20
15
10
5
0
1 hr
0
1
2
3
4
5
6
Hours post s.c. injection in the contralateral foot
mGzmA
mGzmA+Vorapaxar
B
50
Vorapaxar
40
Diluent control treatment
p=0.013
30
% increase foot swelling + SE
20
10
0
0
2
4
6
8
10
12
Day post CHIKV infection
Supplementary Figure 3. (A) Swelling of uninjected contralateral feet. rmGzmA was injected into 6 hind feet of 6 C57BL/6 mice as described in Fig. 3. The mean percentage increase in foot height times width was determined for the contralateral (uninjected) feet. Statistics by Kolmogorov-Smirnov and Kruskal-Wallis tests. Vorapaxar treatment (as in Fig. 3) was also able significantly to reduce swelling of contralateral feet at 0.5 hr. Statistics by Kolmogorov-Smirnov test. I-343 treatment (as in Fig. 3) was also able significantly to reduce swelling of contralateral feet at 1 hr. Statistics by t test. (B) Repeat of Figure 3G. As for Figure 3G except Vorapaxar was dissolved in DMSO. Statistics by t test, n=6 mice per group, the average of right and left foot for each mouse was used.

## Slide 4
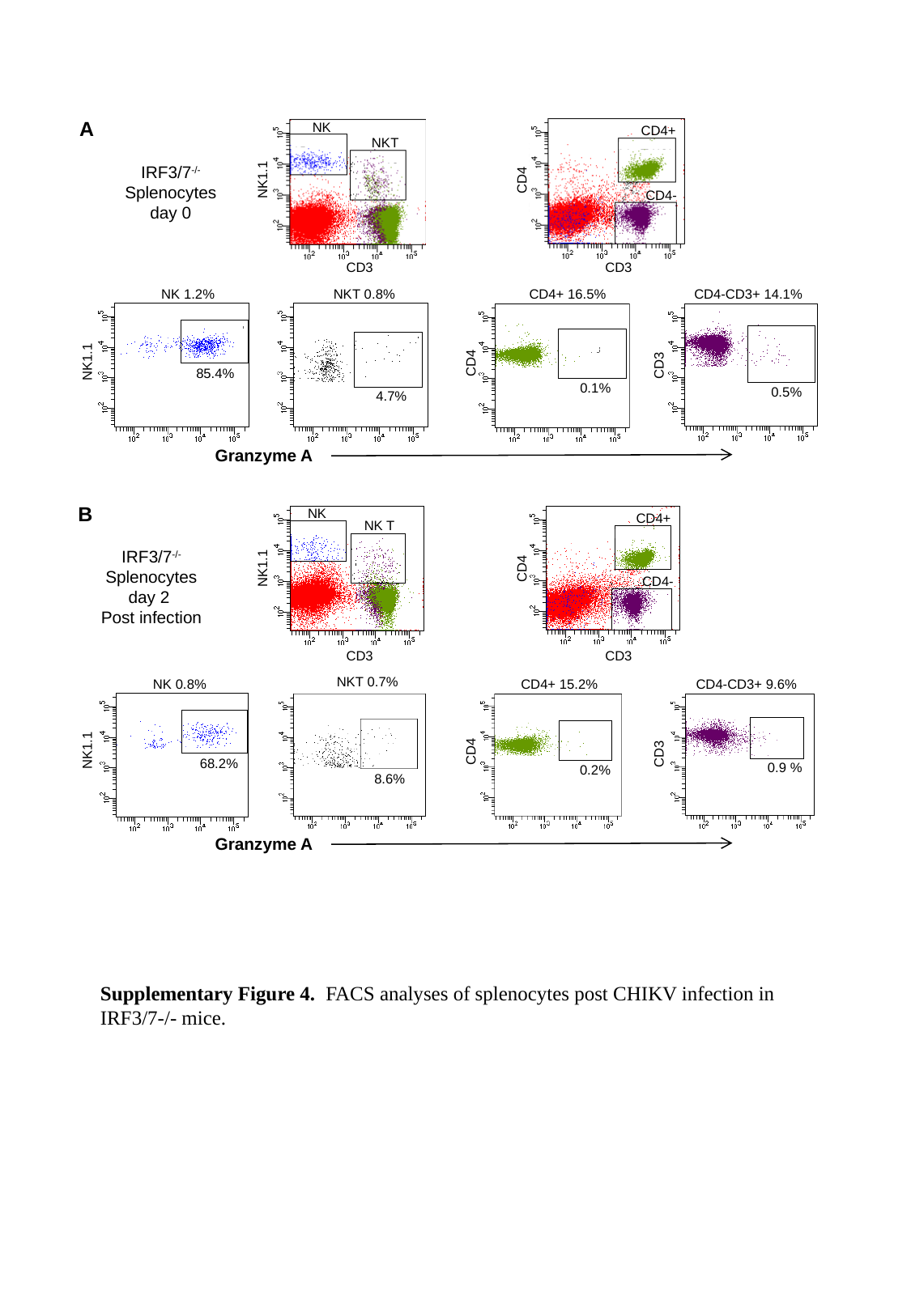

A
NK
NKT
CD4+
CD4-
IRF3/7-/-
Splenocytes
 day 0
NK1.1
CD4
CD3
CD3
NK 1.2%
NKT 0.8%
CD4+ 16.5%
CD4-CD3+ 14.1%
NK1.1
CD4
CD3
85.4%
0.1%
0.5%
4.7%
Granzyme A
B
NK
CD4+
CD4-
NK T
NK1.1
CD4
CD3
CD3
NK1.1
CD4
CD3
Granzyme A
IRF3/7-/-
Splenocytes
day 2
Post infection
NKT 0.7%
NK 0.8%
CD4+ 15.2%
CD4-CD3+ 9.6%
68.2%
0.9 %
0.2%
8.6%
Supplementary Figure 4. FACS analyses of splenocytes post CHIKV infection in IRF3/7-/- mice.

## Slide 5
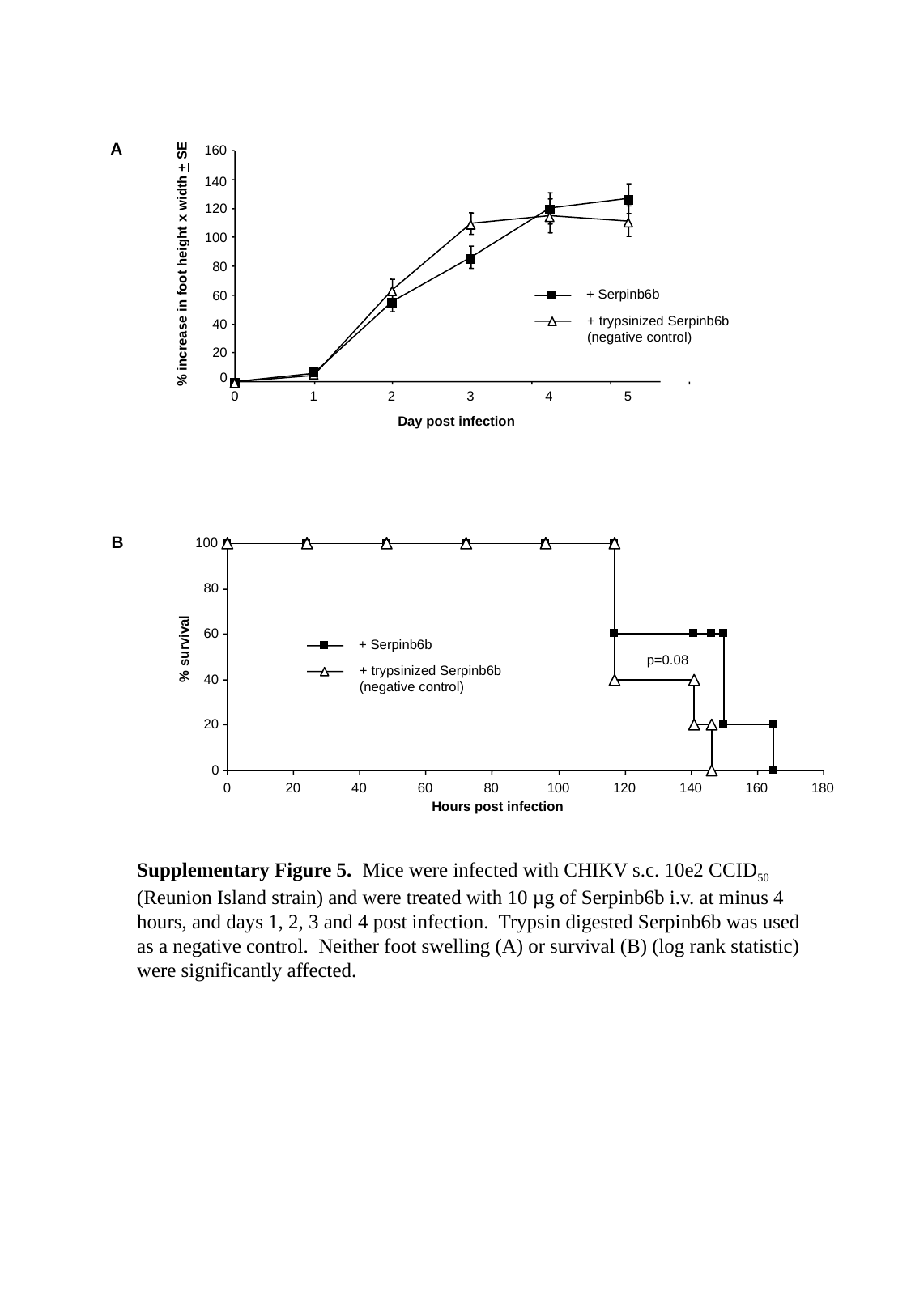

A
160
140
120
100
80
60
40
20
0
% increase in foot height x width + SE
+ Serpinb6b
+ trypsinized Serpinb6b
(negative control)
1
2
3
4
5
0
Day post infection
B
100
80
60
+ Serpinb6b
% survival
p=0.08
+ trypsinized Serpinb6b
(negative control)
40
20
0
0
20
40
60
80
100
120
140
160
180
Hours post infection
Supplementary Figure 5. Mice were infected with CHIKV s.c. 10e2 CCID50 (Reunion Island strain) and were treated with 10 µg of Serpinb6b i.v. at minus 4 hours, and days 1, 2, 3 and 4 post infection. Trypsin digested Serpinb6b was used as a negative control. Neither foot swelling (A) or survival (B) (log rank statistic) were significantly affected.
